# Biomarkers of Inflammatory Bowel Disease

**DOI:** 10.1155/2014/710915

**Published:** 2014-05-19

**Authors:** Yi Fengming, Wu Jianbing

**Affiliations:** Department of Oncology, Second Affiliated Hospital of Nanchang University, Minde Road 1, Nanchang, Jiangxi 330006, China

## Abstract

Inflammatory bowel disease (IBD) is a chronic disease mostly involved with intestine with unknown etiology. Diagnosis, evaluation of severity, and prognosis are still present as challenges for physicians. An ideal biomarker with the characters such as simple, easy to perform, noninvasive or microinvasive, cheap, rapid, and reproducible is helpful for patients and clinicians. Currently biomarkers applied in clinic include CRP, ESR, pANCA, ASCA, and fecal calprotectin. However, they are far from ideal. Lots of studies are focused on seeking for ideal biomarker for IBD. Herein, the paper reviewed recent researches on biomarkers of IBD to get advances of biomarkers in inflammatory bowel disease.

## 1. Introduction

Inflammatory bowel disease (IBD) includes Crohn's disease (CD) and ulcerative colitis (UC) with unknown etiology. Physicians get the diagnosis of IBD usually based on the combination of clinical features, laboratory tests, radiology, endoscopy, and pathology. Diagnosis, evaluation of severity, and prognosis are still present as challenges for clinicians. Laboratory biomarkers are noninvasive or microinvasive, objective, and rapid and cost less than other techniques, which relieve physiological and financial burden for patients. An ideal biomarker for IBD should be simple, easy to perform, noninvasive or microinvasive, cheap, rapid, and reproducible [1]. Unfortunately, there still are no biomarker satisfying these characters. Herein, the authors search “Web of Science” and “Pubmed” by key words “inflammatory bowel disease,” “ulcerative colitis,” “Crohn's disease,” “marker,” and “biomarker” to get advances of biomarkers in inflammatory bowel disease.

## 2. Markers Related to Genetic Predisposition

Family history is a risk factor for developing IBD, with a peak incidence in early adult life, although individuals of any age can be affected [2]. But family history does not affect the severity of CD [3]. Various candidate genes for IBD have been discovered through genome-wide association studies (GWAS) or candidate gene approaches, but only three genetic polymorphisms related to* NOD2*, IL23/17, and autophagy have been well established for a direct pathogenetic role. Furthermore, genetic variants associated with IBD can vary in frequency depending on the cohort ethnicity; it changes with different racial and ethnic groups.

### 2.1. *NOD2*

A large number of identified susceptibility loci have been explored in both CD and UC [2].* NOD2*, the only one contributed to CD risk alone [4]. Homozygous or compound heterozygous mutations in* NOD2* are associated with the reduced activation of transcription factor nuclear factor-*κ*B (NF-*κ*B) [5].

The most common mutation occurs in Caucasians other than eastern Asians. Disease predisposing mutations of* NOD2* are present in Turkish and Iranian patients, but they are absent in Japanese, Han Chinese, Indian, and Malaysian patients with CD [6]. The variants within* NOD2* are mainly predisposed to ileal, stenosing, and familial CD [7]. So sequencing for* NOD2* variants is quite important for Caucasians as it could contribute to CD risk, and it is controversial for Asians.

### 2.2. Autophagy Genes

GWAS for CD shows the genes regulating autophagy, including autophagy 16-like 1 (*ATG16L1*), immunity-related guanosine triphosphatase M (*IRGM*), and leucine-rich repeat kinase 2 (*LRRK2*) genes, which are associated with CD risk [8, 9].

For* ATG16L1*, studies show that 12 previously CD-associated SNPs in West are not found in Asians studied [10]. Moreover, 8 SNPs of the* IRGM* gene did not show relation with CD and UC in a Japanese study [11]. However, a Korean study shows that* IRGM* SNP rs10065172 is significantly associated with CD susceptibility and also find a protective relationship between the SNP rs72553867 and the CD susceptibility [12]. So it is confused with different races, and more researches are needed to confirm it.

### 2.3. *IL23R*

Several common variants in the IL-23 receptor gene (*IL23R*) are reported to be clearly associated with both CD and UC susceptibility [10]. But in east Asia,* IL23R* variants does not show any association with CD [13–15].* IL23R* is a CD susceptibility gene, but different* IL23R* variants are likely to carry variable disease-modifying effects in different populations.

The gene also affects the strategies of treatment. A research in Germany shows that homozygous carriers of IBD risk-increasing* IL23R* variants are more apt to respond to anti-TNF than homozygous carriers of IBD risk-decreasing* IL23R* variants [16].

## 3. Markers Related to Disease Type

IBD is an immune-related disease, some immune-associated markers are also explored for this disease. The differentiation of UC and CD is also quite difficult for physicians especially when the clinical, endoscopic, and pathologic features are not typical or confused. However, some markers could help to resolve part of them.

### 3.1. Antineutrophil Cytoplasmic Antibodies

Antineutrophil cytoplasmic antibodies (ANCAs) are antibodies for granules of neutrophil cytoplasm; it is first reported in UC patients in 1990 [17]. Atypical perinuclear ANCA (pANCA) is DNase sensitive [18]; it increases significantly in UC [19]. A prospective followup study recruiting 197 IBD-unclassified (IBD-U) demonstrates that 64% UC patients is anti-*Saccharomyces cerevisiae* antibody (ASCA)−/pANCA+ [20]. Another nation-based survey shows that the positive rate of pANCA is 55% in UC, 48% in rheumatoid arthritis, and 32% in healthy people [21]. We recruit 152 UC, 54 CD, and 60 IBD-U demonstrating that the sensitivity and specificity of pANCA are 43.3% and 96.3% separately when compared to healthy controls (HC) [22].

### 3.2. Anti-*Saccharomyces cerevisiae* Antibodies

ASCAs are antibodies for mannan in cell wall of* Saccharomyces cerevisiae* (*S. cerevisiae*); it is homologous to cell wall of enterobacterias [23]. Mallant-Hent et al. find that ASCA does not exist in membrane of* S. cerevisiae*, which indicates that ASCAs have no relationship with mucosa exposure of* S. cerevisiae* [24]. ASCAs have best sensitivity and specificity, it could reach up to 31%–45% and 90%–100%, respectively, when compared with other antibodies such as anti-*Escherichia coli* outer-membrane porin C (anti-OmpC), anti-chitobioside carbohydrate IgA antibodies (ACCAs), anti-laminaribioside carbohydrate IgG antibodies (ALCAs), anti-mannobioside carbohydrate IgG antibodies (AMCAs), anti-chitin (anti-C), and anti-laminarin (anti-L) [19]. A prospective long-term followup study including 197 IBD-U shows that 80% CD are ASCA+/pANCA− [20]. The sensitivity and specificity for ASCA in CD are 46.3% and 96.3%, respectively [22]. Another research admits 15 idiopathic ocular inflammations without IBD but with IBD family history; pANCA increases in 8 patients, however it increases in just 3 healthy controls (*P* = 0.004) [25].

Apart from pANCAs and ASCAs, other serological antibodies such as anti-OmpC, ALCAs, ACCAs, AMCAs, anti-L, and anti-C and pancreatic autoantibodies (PAB) also contribute to diagnosis and differential diagnosis of IBD and other diseases [19, 26–28].

## 4. Markers Related to Inflammation or Disease Activity

Various markers have been proposed to objectively evaluate disease activity or inflammation, but sensitivity and specificity have been a concern for each. A combination of biomarkers may be the most useful for prediction or confirmation of clinical disease activity and endoscopically visible inflammation.

### 4.1. C-Reactive Protein and Hypersensitive C-Reactive Protein

C-reactive protein (CRP) is considered as one of the most important protein in acute inflammation; it is consist of 5 components [29]. It maintains low level in circulation secreted by hepatocytes in healthy individuals (<1 mg/L), but it sharply increases even reaching up to 350–400 mg/L when acute inflammation which is induced by interleukin-6 (IL-6), tumor necrosis factor-*α* (TNF-*α*), and IL-1*β*. The level 10–40 mg/L indicates chronic inflammation or virus infection. Half-life of CRP is quite short (19 h); it increases rapidly and decreases sharply in acute inflammation. It responsds differently in UC and CD; CD is correlated with CRP significantly but UC is not [30, 31]; the mechanism is still unknown. Hypersensitive C-reactive protein (Hs-CRP) and *β*2-microglublin correlated with histology scores of UC [32]. Hs-CRP is useless in evaluation of disease severity and corticosteroids therapy of pediatric IBD with normal level of CRP [33].

### 4.2. Erythrocyte Sedimentation Rate

Erythrocyte sedimentation rate (ESR) indicates migration speed of red blood cells in plasma; it varies with concentration of plasma and the size of erythrocyte. It is significantly altered when in patients with anemia, globulism, or Mediterranean anemia [34]. Time to peak and decline is delayed for ESR when compared with CRP.

### 4.3. Platelets Count

Platelets (PLT) increase in patients with IBD, which contribute to high-coagulated state of IBD, such as the formation of microthrombus [35–37]. Moreover, reticulated platelet levels increase significantly in patients with ulcerative colitis [38].

### 4.4. Mean Platelet Volume

Mean platelet volume (MPV) indicates average size of platelet; it could reflect the rate of platelet stimulation and production. Kapsoritakis et al. [39] find out that MPV decreased significantly in active-IBD, and it negatively correlated with some markers of inflammation, such as white blood cell (WBC), CRP, and ESR. However, another research does not show any relationship between this parameter and disease activity [40]. The decline of MPV may be related with disturbance of thrombus formation when inflammation occurs [39].

### 4.5. Red blood Cell Distribution

Red blood cell distribution (RDW) reflects the size and variability of erythrocytes in peripheral circulation, it is usually detected in blood regular test in hospital [41]. A study including 221 IBD (120 UC and 101 CD) shows that CRP, ESR, and RDW increase in IBD (*P* < 0.05); multivariate analysis shows that RDW has the best prediction when evaluated disease activity for CD patients with nonanemia. When the cutoff of RDW was 13.8, the sensitivity and specificity are 76% and 86% for nonanemic UC separately; it could reach up to 82% and 83% for nonanemia CD when cutoff was 14.1 [42]. Another study demonstrates reticulocyte distribution width (RDWR) and red blood cell size (RSF) have significant diagnostic value for IBD with iron-deficiency anemia; it could reflect disease activity and anemia for IBD patients [43].

### 4.6. Fecal Calprotectin

Fecal calprotectin is a protein in neutrophil granulocytes and macrophages; it consists of S100A8 and S100A9 and is first found and described in 1980 [44]. It is stable and well-distributed in feces which make it reflect the entire state of the feces when we detect a part of it [45]. Calprotectin is considered as one type of damage associated with molecular pattern protein (DAMP). It is released by activated innate immunity cells when cell stresses and damages, which also reflect the process of inflammation [46].

More and more studies focus on fecal calprotectin in IBD and confirm its value in diagnosis, disease activity evaluation, effect evaluation, and relapse monitor [46]. A prospective cohort study demonstrates that the sensitivity and specificity of CD are 100% and 97% (cutoff 30 mg/L) when compared to irritable bowel syndrome (IBS) [47].

Schoepfer et al. detect fecal calprotectin and IBD-related antibodies; they show that the accuracy of fecal calprotectin is 89% when compared to IBS (sensitivity and specificity were 83% and 100%, resp.); the accuracy is just 91% when combined with ANCAs or ASCAs [48].

A meta-analysis including 30 prospective studies confirms that the sensitivity and specificity of fecal calprotectin could reach up to 95% and 91%, respectively; the accuracy in children is higher than in adults. Moreover, fecal calprotectin is better than CRP, ESR, ASCA, pANCA, and OmpC [49]. Another meta-analysis shows that fecal calprotectin could reduce 67% of endoscopy in adult, but it also could delay the treatment of 6% of patients as its false negative [50]. Studies also find fecal calprotectin level is significantly higher in active colonic CD than in active ileal CD [51]; left-sided and distal UC are higher than pancolonic UC [52]; fecal calprotectin also could evaluate the effect of treatment [53]. Calprotectin decreases significantly after IFX treatment for 12 weeks, and it correlated with endoscopic index of severity (CDEIS) (*r* = 0.561, *P* = 0.03) [54]. Røseth et al. show that fecal calprotectin level correlated with endoscopic mucosal healing [55]. A meta-analysis focusing on fecal calprotectin in IBD relapse shows that the sensitivity and specificity when predicting the relapse are 78% and 73%, separately [56]. A study recruiting 60 newly diagnosed CD patients without treatment finds there is no difference of calprotectin level between small intestine involved only and small intestine and colon involved; it is not correlated with pediatric CD activity index (CDAI), but it decreases as other markers such as ESR or CRP [57]. Calprotectin in 1/3 of the children decreases significantly when treated with IFX for pediatric IBD, which indicates that it gets mucosal healing [58]. A study demonstrates that calprotectin level >100 mug/g could implicate positive findings in capsule endoscopy; there is no need of capsule endoscopy when <100 mug/g [59]. The use of fecal calprotectin would improve diagnostic yield in patients with abdominal complaints in addition to European Panel on the Appropriateness of Gastrointestinal Endoscopy (EPAGE) criteria [60].

A systematic review conclude that, for distinguishing between IBD and IBS in adults, these gave pooled sensitivity of 93% and specificity of 94% at fecal calprotectin cutoff  level of 50 *μ*g/g. Sensitivities at that cutoff ranged from 83% to 100% and specificities from 60% to 100%. For distinguishing between IBD and non-IBD in pediatric populations with ELISA tests, sensitivities ranged from 95% to 100% at cutoff of 50 *μ*g/g and specificities of 44%–93%. Fecal calprotectin can be a highly sensitive way of detecting IBD, although there are inevitably tradeoffs between sensitivity and specificity, with some false positives (IBS with positive calprotectin), if a low calprotectin cutoff is used. In most cases, a negative calprotectin rules out IBD, thereby sparing most people with IBS from having to have invasive investigations, such as colonoscopy [61].

National Institute for Health and Care Excellence (NICE) guideline for fecal calprotectin diagnostic tests for inflammatory bowel disease gives recommendations for the differential diagnosis of inflammatory bowel disease (IBD) or irritable bowel syndrome (IBS) in adults or children, and it is a useful screening test to triage referrals to gastroenterology for investigation of chronic diarrhea, without rectal bleeding, in patients under the age of 60 years [62].

### 4.7. Fecal Lactoferrin

Lactoferrin is an iron-binding protein; it covers most mucosal surface and interacts with exocrine organs or substances including parotid, tears, vaginal discharge, articular synovia, and latex [63–65]. It is a component of neutrophil granulocytes and activated in acute inflammation [65]. Fecal lactoferrin increases significantly as infiltration of neutrophils in intestinal tracts [66]. It is stable for 5 days in feces whenever repeating freeze thawing [67].

Sugi et al. find that lactoferrin could reflect inflammation of intestine [68]. The diagnostic rate of lactoferrin for IBD could reach up to 80% when compared with IBD, which is similar to fecal calprotectin and better than CRP (64%) [69]. The sensitivity and specificity are 86% and 100% when compared with HC and IBS, and there is a significant difference between active-IBD and inactive-IBD [65], but a study does not find any difference between active-CD and inactive-CD [70]. Fecal lactoferrin also associated with disease activity and ESR pediatric IBD patients [67].

### 4.8. Fecal Neopterin

Both fecal calprotectin and fecal neopterin concentrations correlated with endoscopic scores in UC (*r* = 0.75 and *r* = 0.72, resp.; *P* < 0.0001 for both) better than in CD (*r* = 0.53 and *r* = 0.47, resp.; *P* < 0.0001 for both). Using cutoffs of 250 *μ*g/g for fecal calprotectin and 200 pmol/g for fecal neopterin, both fecal markers have similar overall accuracies to predict endoscopic activity in patients with CD (74%) and a higher accuracies in patients with UC (88% and 90%, resp.), whereas accuracies of C-reactive protein are slightly lower in patients with CD and UC [71]. Another study recruiting 70 CD and 52 UC shows fecal neopterin increases just in feces other than in serum and urine [72].

### 4.9. S100A12

Scholars consider S100A12 as a novel biomarker which could meet these characters: high sensitivity and specificity, easy to take and detect, cheap, and of good compliance [73]. S100A12 is a member of S100 calcium binding protein family [74]; it is activated extracellular similar to S100A8 and S100A9. S100A12 participates a lot in proinflammation processes; it stimulates proinflammation mediators by NF-*κ*B or other similar pathways [75]. It could be stable for 7 days in room temperature and increases in inflammatory diseases such as arthritis and Kawasaki disease [76, 77]. It also correlated with the prognosis of severe respiratory reaction in premature children [78]. Early diagnosis and inflammation control seem to be quite important for pediatrics; it could avoid long-term or short-term complications. Uncontrolled inflammation usually correlated with loss of weight, restriction of height, and delay of adolescence [79]. Fecal S100A12 correlated with inflammatory biomarkers and pediatric CD activity index (pCDAI) [80]. Fecal S100A12 decreases gradually when the children got clinical remission [80]. However, S100A12 increases significantly in severe acute pediatric UC patients, but it does not correlated with response of treatment [81].

The sensitivity and specificity of fecal S100A12 could reach up to 86% and 96% respectively, it is higher than fecal calprotectin [82]. Another study also shows S100A12 is better than calprotectin when compared with HC; its sensitivities in CD and UC are 81% and 91% separately, and the specificity is 100% in both groups. Fecal S100A12 is also elevated in bacterial enteritis but not in viral gastroenteritis. Fecal S100A12 correlated better with intestinal inflammation than fecal calprotectin or other biomarkers [82].

Foell et al. shows that S100A12 upregulated in serum of UC and CD, and it correlated with disease activity [83]. Studies also demonstrate the level of fecal S100A12 decreases as anti-inflammation treatment, suggesting that this marker could reflect the response of drug treatment. S100A12 could predict mucosal healing and recurrence of disease; the method of evaluation of mucosal healing currently is endoscopic and histopathological examination; the invasive, and expensive examinations could be replaced by noninvasive detection [73]. However, more studies need to confirm S100A12 in IBD evaluation.

A study aims to investigate the role of calprotectin and S100A12 in predicting inflammatory lesions of small bowel in patients undergoing wireless capsule endoscopy (WCE). The result shows that fecal calprotectin is significantly higher in CD patients compared with those with normal WCE or other abnormalities (*P* = 0.008), whereas fecal S100A12 does not differ between the groups. When detecting inflammatory small bowel lesions, sensitivity, specificity, positive predictive value, and negative predictive value for fecal calprotectin (cutoff 50 *μ*g/g), they are 59%, 71%, 42%, and 83%, and for S100A12 (cutoff 0.06 *μ*g/g), these are 59%, 66%, 38%, and 82% [84]. A review concludes that S100A12 is valuable in diagnosis, distinguish, recurrence monitor, and treatment of IBD [85].

### 4.10. MicroRNAs

MicroRNAs (miRNAs) are small noncoded single-stranded RNAs (approximately 18–24 nucleotides); they are negatively regulatory molecules for genes. The unbalance of miRNAs could emerge in physiopathologic processes of multiple diseases, such as alloplasia, arrhythmia, schizophrenia, cancer, and immune-related diseases [86]. The effect of miRNAs in innate immunity and adaptive immunity such as immune-mediated psoriasis, rheumatoid arthritis, asthma, and systemic lupus erythematosus (SLE) has been confirmed by studies [87]. Some serological miRNAs upregulated or downregulated in IBD [88].

### 4.11. Adenosine Deaminase

There is significant increase of adenosine deaminase (ADA) in active CD patients when compared with HC and patients in remission, and ADA correlated with CRP (*r* = 0.516), which may be a new biomarker for CD activity [89].

### 4.12. Lipopolysaccharide-Binding Protein and CD14

A research recruits 214 CD and 110 HC, to detect lipopolysaccharide-binding protein (LBP), CD14, hs-CRP, ASCA IgG/IgA, anti-OMP IgA, and pANCA. Serological LBP increases and soluble CD14 decreases significantly. In addition, LBP and CD14 correlated with hs-CRP; the accuracy of evaluation of active CD equals to hs-CRP [90].

### 4.13. Abnormal Lectin-Based IgG Glycosylation

Serological IgG in IBD has higher affinity for lectin compared with HC. A study takes in 410 IBD (include 290 Japanese and 161 Americans) and HC; abnormal lectin-based IgG glycosylation increases significantly in CD when compared with HC; it correlated with disease activity and has higher specificity than CRP when it combines with ASCA [91].

### 4.14. Mopterin

Mopterin is a component of piperazine-[2,3-d]-pyrimidine, which is a metabolic product of cyclic guanosine monophosphate [92]. Mopterin is released by* T* lymphocytes and macrophages stimulated by *γ*-interferon [93]. Some researchers find that mopterin increases in urine and serum of UC and CD [92, 94–97]. Another study shows tumor necrosis factor-*α* (TNF-*α*) correlated with serological mopterin (*r* = 0.73, *P* < 0.0001) [98]. Serological mopterin also correlated with ESR [97]. However, a study including 70 CD and 52 UC does not find any changes of mopterin in serum and urine but rather in stool [72].

### 4.15. Soluble ST2

ST2 is a member of IL-1R superfamily; it is consist of 2 parts (ST2L and sST2) and coded by 2nd chromosome [99]. A study recruits 110 IBD (82 UC and 26CD) and healthy controls shows that serological sST2 is higher in active-UC when compared with nonactive ones; the sensitivity, specificity, and accuracy are 83.3%, 83.3%, and 83.3%, respectively. Moreover, it significantly correlated with endoscopic activity scores [100].

### 4.16. Nitric Oxide

A study (30 UC, 30 CD, and 30 HC) demonstrates that there are significantly different concentrations of nitric oxide (NO) between three groups. With a cutoff level of 17.39 *μ*mol/L NO has a sensitivity of 100% and a specificity of 100% in distinguishing active and inactive UC patients. With cutoff value of 14.01 *μ*mol/L serum NO level has a sensitivity of 88% and a specificity of 69% in distinguishing patients with active CD and inactive CD [101].

Exhaled nitric oxide (NO) could indicate Crohn's disease involved in lungs; a study shows that exhaled NO increases significantly in CD compared to controls [102].

### 4.17. Soluble Triggering Receptor Expressed on Myeloid Cells-1

Soluble triggering receptor expressed on myeloid cells-1 (sTREM-1) notably correlated with endoscopic activity score, which indicates it might be a biomarker to evaluate endoscopic activity [103].

### 4.18. Substance P

Serum substance P level sharply increases in 61 UC and 66 CD when compared with HC (*P* < 0.01); it correlated with disease activity in UC (*P* = 0.014) and it decreases immediately when get endoscopic and clinic remission (*P* = 0.025). However, there is no difference between active CD and inactive CD, it usually increases in inactive stricture or penetrate CD [104].

### 4.19. Activated Thrombin Activatable Fibrinolysis Inhibitor

Activated thrombin activatable fibrinolysis inhibitor (TAFIa) is considered to be related with pathogenesis and progress of IBD. Plasma TAFIa increases significantly in CD, and it correlated with WBC, CRP, fibrinogen, and platelet, which indicates it might be a potential IBD biomarker [105].

### 4.20. Quantitative Fecal Immunochemical Test

Quantitative fecal immunochemical tests (FITs) could detect fecal blood rapidly. A study consists of 152 UC demonstrates that the sensitivity and specificity of FIT could reach up to 92% and 71% when cutoff <100 ng/mL; FIT also correlated with Mayo scores [106].

### 4.21. Chitinase 3-Like-1

Chitinase 3-like-1  (CHI3L1/YKL-40) is a protein excreted by endotheliocytes and macrophages in intestine. The sensitivity and specificity of fecal CHI3L1 could reach up to 84.7% and 88.9% when cutoff was 13.7 ng/g in active-IBD [107].

### 4.22. Angiogenin

Angiogenin increases significantly in IBD when compared with HC. Moreover it shows a sharply decrease of agiogenin when treated IBD patients with aminosalicylic acid (ASA), which indicates that angiogenesis is unbalance in IBD patients [108].

### 4.23. Mucosal Indoleamine 2,3 Dioxygenase-1

The activity of mucosal indoleamine 2,3 dioxygenase-1 (IDO1) is quite important in IBD diagnosis [109].

### 4.24. Mucosal Cytokine

Higher levels of IL-17A and IFN-*γ* are significantly correlated with remission after three IFX infusions (OR = 5.4, *P* = 0.013 and OR = 5.5, *P* = 0.011, resp.). IL-17A and IFN-*γ* mRNA expression showed positive correlation. Th2 and Treg-related mediators are not significantly associated with clinical outcome but are expressed at higher levels in UC patients when compared with the controls. High expression of Th1- and Th17-related cytokines could potentially predict a favorable outcome of IFX induction therapy in the mucosa of UC patients. Th2 and Treg-related mediators do not appear to be useful as predictive markers [110].

Mucosa-related biomarkers such as mucosal CD11c+ [111], cytokines and chemotactic factors, adhesion molecules, et al., are far from being ideal biomarkers [112].

### 4.25. Urine Neopterin

Neopterin in urine is used to distinguish active state and nonactive ones [92]. However, another study does not find any change for urine neopterin in different state of the disease [72].

Others such as soluble intercellular adhesion molecule-1, D-lactate, diamine oxidase,* Pseudomonas fluorescens* CD-related protein, OmpC, and CBir1 flagellin also correlated with IBD [113–115]. Dipeptidyl peptidase-4 (DP4) decreases significantly in CD which also might be a potential biomarker [116].

## 5. Markers Related to Drug Metabolism

Drugs for inflammatory bowel disease include aminosalicylic acid, corticosteroids, immunosuppressant, and anti-TNF agents. However, the reaction rate for patients with IBD is not ideal. Some markers are proposed to monitor the effect of the drugs.

### 5.1. Antibodies toward Infliximab

As biological agents are widely used in IBD, effect-monitoring of biological agents seems to be quite important. Serological antibodies to infliximab (ATIs) usually affect the treatment of infliximab (IFX). A meta-analysis collecting 13 studies including 1378 IBD patients shows the risk ratio (RR) of patients with ATIs failed treatment was 3.2 (95% CI: 2.0–4.9, *P* < 0.0001) when compared with non-ATIs or low-ATIs. Although there exists a bias in this study, the research recently shows ATIs concentration significantly correlated with the effect of treatment, so the concentration of ATIs seems to be quite important for patients with IFX [117, 118]. Furthermore, the antibodies to F(ab′)2 exist in 67% ATI-positive patients; it is immunogenicity of ATI, but scholars declare ATIs are still more important than F(ab′)2 when there is effect-monitoring of IFX in IBD [119].

pANCA seems to be valuable for predicting response to anti-TNF, as negative status of pANCA is associated with early response to anti-TNF drugs [16]. Th1- and Th17-related cytokines could potentially predict a favorable outcome of IFX induction therapy in the mucosa of UC patients. Th2 and Treg-related mediators do not appear to be useful as predictive markers [110].

### 5.2. Thiopurine Methyltransferase and 6-Thioguanine Nucleotide

The activity of thiopurine methyltransferase (TPMT) and the concentration of 6-thioguanine nucleotide (6TGN) are considered to be related with the treatment of thioguanine. However, recent study does not find any relationship between them [120].

### 5.3. Urine Salicylate Level

A study including 93 patients with UC taking mesalamine maintenance therapy prospectively enrolled from the clinics. Random urine salicylate levels (by colorimetry) were highly correlated with urine 5-ASA metabolite levels (by mass spectrometry; *R* ^2^ = 0.9). A random urine salicylate level above 15 mg/dL distinguishes patients who have recently taken mesalamine from controls (area under the curve value 0.9, sensitivity 95%, and specificity 77%) [121].

### 5.4. Genes

Some studies also found the association between genetic factors and response to treatment; they affect the strategies of treatment. Multidrug resistant 1 (*MDR1*) polymorphisms is associated with corticosteroid refractoriness in CD and UC, and it is also correlated with a higher risk of cyclosporine failure in patients with steroid-resistant UC [122, 123]. A research in Germany shows homozygous carriers of IBD risk-increasing* IL23R* variants are more apt to respond to anti-TNF than homozygous carriers of IBD risk-decreasing* IL23R* variants [16]. Another study such as apoptosis genes polymorphisms predict response to infliximab therapy in luminal and fistulizing Crohn's disease [124].

## 6. Markers Related to Neoplastic Transformation

Ulcerative colitis is considered to be a precancerous lesion; it could progress to colorectal cancer (CRC) as the disease duration extends. So the surveillance for it before canceration should be emphasized.

### 6.1. M2-Pyruvate Kinase

M2-pyruvate kinase (M2PK) often exists in undifferentiated or proliferous tissues and could be detected in serum and feces [125]. It increases in multiple traumas, chronic cardiac failure, tumors, and pouchitis [126–129], some scholars also find the relationship between M2PK and IBD [130]. The enzyme was stable in room temperature and could be detected by enzyme linked immunosorbent assay (ELISA) [129]. M2PK increases significantly in UC, CD, and CRC when compared with IBS, and it is lineararily correlated with calprotectin. It might be a potential marker for screening CRC in IBD patients, and more researches need to confirm it.

### 6.2. miRNAs

Studies found miRNAs up-regulated or down-regulated in mucosa of different sites [131, 132]. PDCD4/miR-21 was often un-balance in precancerous changes or intraepithelial neoplasias of mucosa in IBD. miR-21 increases but PDCD4 decreases may indicate they could be biomarkers of IBD canceration [133]. miRNAs increases significantly in mucosa of IBD by micro-array analysis and real-time polymerase chain reaction [134].

There is no literature on fecal miRNAs and IBD, but fecal miRNA-92a was confirmed to be predictive in CRC and adenomatous polyp; the sensitivities were 71.6% and 56.1%, respectively; specificities were both 73.3% [135].

### 6.3. Mucosal CHI3L1

Mucosa CHI3L1 is highly expressed in intraepithelial neoplasias mucosa of UC cryptoepithelium and increases when developed to CRC. So it might be a biomarker to monitor malignant degeneration of UC [136].

## 7. Conclusions

Studies on IBD biomarkers were subdivided into 5 parts; however, invasive, expensive, physical, and mental burden for mucosa or tissue restricts its applications. Currently biomarkers applied in clinic include CRP, ESR, pANCA, ASCA, and fecal calprotectin; other biomarkers still need to be confirmed in large clinic research. These biomarkers bring convenience for patients and clinicians, but biomarkers are still far from ideal.
